# The changing relationship between rainfall and children’s physical activity in spring and summer: a longitudinal study

**DOI:** 10.1186/s12966-015-0202-8

**Published:** 2015-03-21

**Authors:** Flo Harrison, Esther MF van Sluijs, Kirsten Corder, Ulf Ekelund, Andy Jones

**Affiliations:** Norwich Medical School & UKCRC Centre for Diet and Activity Research, University of East Anglia, Norwich, UK; MRC Epidemiology Unit & UKCRC Centre for Diet and Activity Research (CEDAR), University of Cambridge School of Clinical Medicine, Institute of Metabolic Science, Cambridge Biomedical Campus, Box 285, Cambridge, CB2 0QQ UK; Department of Sports Medicine, Norwegian School of Sports Sciences, Ullevål Stadion, Oslo, Norway

**Keywords:** Physical activity, Weather, School, Children, Rainfall

## Abstract

**Background:**

Weather conditions, along with day length, are proposed as the main drivers of the seasonal patterns in children’s physical activity (PA), but little is known about how they affect children at different ages. This study examines the relationship between rainfall and PA in a longitudinal cohort of initially 9–10 year-old children in Norfolk, UK.

**Methods:**

Participants were 283 children from the SPEEDY study who wore accelerometers ≤7 days on three occasions in the summer of 2007, 2008 and 2011 at ages 9–10, 10–11, and 13-14y. Daily weather data were obtained for two local weather stations. Relationships between rainfall and PA (moderate-to-vigorous-PA (MVPA; ≥2000) vigorous PA (VPA; ≥4000), counts per minute (cpm)) and sedentary time were assessed in multiple-membership multilevel models. PA was assessed over the whole day, and over parts of the school day; commute time (8 am-9 am and 3 pm-4 pm), lunchtime (12noon-2 pm), and after school (4 pm-9 pm).

**Results:**

At ages 9–10 and 10-11y, PA declined with increasing rainfall, with an average of 14.0 (SE 2.9) and 11.4 (3.0) minutes less MVPA on the wettest days (≥1.7 mm rain) compared to dry days respectively. There was no significant trend in MVPA across rainfall categories at age 13–14 years. Between ages 9–10 and 13–14, MVPA decline was largest on dry days (-15.2 (2.7) minutes). These patterns were also apparent during school lunchtime and after school, however they were not seen during school commute times. Similar patterns were seen for other PA intensities.

**Conclusions:**

Increased rainfall is associated with significant decreases in PA among primary school children, but not secondary school children. PA declines most steeply between the ages of 9–10 and 13–14 on dry days. Interventions to increase activity on wet days may be most relevant at primary schools. Our results also highlight the importance of habitualising behavior to make children more resilient both to bad weather, and potentially age-related decline in activity.

**Electronic supplementary material:**

The online version of this article (doi:10.1186/s12966-015-0202-8) contains supplementary material, which is available to authorized users.

## Introduction

Children’s physical activity in the United Kingdom, and other countries at middle to high latitudes, is seen to exhibit a seasonal pattern [1-5]. It is suggested that this pattern is predominantly driven by changes in day length and weather conditions, particularly as activity levels are lowest in the winter when days are shorter, colder and wetter [6]. Rainfall in particular has been identified as the predominant weather related barrier to being physically active year-round [7], operating primarily by inhibiting outdoor play [8], which is typically of a higher intensity [9]. A clear understanding of this behavioural driver may help in the appropriate targeting of interventions to increase physical activity at times of the year and in weather conditions currently associated with low engagement. Consideration of the impacts of weather on children’s physical activity may also lead to an improvement in the design of environmental interventions so that they work well in all conditions. Examples include the provision of sheltered outdoor spaces, or ground covers not subject to water-logging.

Schools are important settings for interventions to promote children’s physical activity. Through commuting, break times, and physical education lessons they provide regular opportunities for children to be active [10] and alterations and additions to the physical school environment have been seen to increase children’s activity levels [11]. However, little work has considered how more changeable aspects of the environment, such as weather, impact school-based physical activity, and the success and sustainability of interventions in all conditions has not been assessed.

We have previously observed a relationship between rainfall and children’s physical activity in the summer months among primary school children [12], possibly suggesting that more attention should be paid to promoting or enabling physical activity in wet weather in primary school settings. However we do not know if and how this relationship may change as children age, and whether such considerations may be as important in secondary schools. In addition to enabling better targeted interventions, there are also potential methodological implications in understanding how seasonal environmental exposures impact behavior, as they may limit the comparability of physical activity measurements taken in different settings or at different times. Such comparisons could be particularly problematic if daily weather conditions strongly impact physical activity, and if this impact differs across age groups.

Using physical activity measurements taken at three time points in the “Sport Physical activity and Eating behaviours: Environmental Determinants in Young people” (SPEEDY) cohort, this study investigates how the relationship between summer rainfall and children’s physical activity changes as they age.

## Methods

### Recruitment and data collection

The SPEEDY study (Sport, Physical activity and Eating behaviour: Environmental Determinants in Young people) is a population based longitudinal cohort study designed to investigate factors associated with diet and physical activity behaviour of children across the county of Norfolk, UK. The study’s methods are described in detail elsewhere [13,14] and so are only briefly recounted here.

In 2007, schools across Norfolk with at least 12 Year 5 pupils (age 9/10 years) were sampled according to a stratification by urban/rural status [15]. After a pilot study among 33 children at one school, 91 schools took part in the main study, and 2031 children were recruited. Baseline data collection was performed during the school summer term (April to July). Teams of trained Research Assistants performed measurements at participating schools according to standard operating procedures. Participant height and weight were recorded using a Leicester height measure and non-segmental Tanita scales (type TBF-300A). Participants were fitted with an accelerometer (Actigraph GT1M) and were given a pack to take home including a questionnaire for their parents or carers to complete.

Participants were invited to undertake further physical activity measurements at +1 year, when aged 10/11y in Year 6, the final year of primary education, and again at +4 years when aged 13/14y in Year 9, the third year of secondary education. Measurement at age 10–11 years focused on physical activity, and at age 13–14 years the full suite of study measures (physical activity, anthropometry and questionnaires) were repeated. Follow-up phases were undertaken during the school summer terms of 2008 and 2011.

### Physical activity measurement

Accelerometers were set to record at 5 second epochs. Participants were asked to wear the devices on their right hip for seven days, removing them overnight and for aquatic activities. The first day of data collection was removed from all files, and 10 minutes of continuous zero counts were classified as ‘non-wear time’. ‘Wear time’ was derived by subtracting minutes of ‘non-wear time’ from the total minutes in a given period. For the analyses presented here, days for which there were fewer than 500 minutes of wear time were excluded, as were children who recorded less than one valid school day of physical activity measurements on all three measurement occasions. These exclusion criteria are in line with the study’s protocol [13,14].

For each valid measurement day, four physical activity outcomes were calculated; mean counts per minute (cpm), time spent sedentary (SED; <100 cpm), time spent in moderate-to-vigorous intensity physical activity (MVPA; >2000 cpm), and time spent in activity of vigorous intensity (VPA; >4000 cpm). The threshold of 2000 cpm is equivalent to walking at 4 km/h [16] and has been used to define MVPA previously in this study [13,17] and others [18].

### Rainfall

Local weather data were obtained from the UK Meteorological Office [19]. Two stations in Norfolk were identified as having near continuous data for the whole study period: Marham, in the southwest of the county, and Weybourne on the north coast (50 km apart). Both stations reported hourly measurements of rainfall, and daily records of temperature. For these stations we extracted hourly rainfall (mm), and summed values to provide daily (7 am to 9 pm) totals. There was strong correlation between the rainfall recorded at the two stations (r = 0.734, p < 0.001), so when data were available from both we calculated the mean value (data from both stations were available for 97% of the study period), otherwise data from one station was used. Both weather measures were linked by date to the physical activity records. For ease of presentation rainfall was banded into three categories; days with no rain (52% of measurement days), and days with 0.1-1.6 mm of rain and those with > =1.7 mm. This latter threshold was determined based on the median of rainfall across all days with rain over the three study waves. Given the absence of physical activity-specific rainfall thresholds, these cut-points were guided by the distribution of rainfall data across all measurement days so as to ensure as even a distribution of physical activity measurements across the categories as possible.

### Statistical analysis

As the SPEEDY sampling frame was based on schools, relationships between rainfall and each physical activity outcome were assessed in multiple membership multilevel regression models. These models take into account the hierarchical nature of the sample of measurement days nested within children, nested within schools. As children from any given primary school attended several different secondary schools, and each secondary school has taken in pupils from several different primary schools, a simple multilevel model of days nested within children nested within schools would not adequately model the hierarchy in our data. The multiple membership component of the model accounts for clustering of children at both primary schools and secondary schools separately. Age, sex, accelerometer wear time (for SED, MVPA and VPA models), mean daily temperature and day type (weekend/week day) were included as covariates whilst rainfall category and study phase were fitted as main effects and as a paired interaction. So that trends could be examined, the models produced were used to predict physical activity at each study phase/rainfall category at the mean values of other covariates.

Models were fitted for all days considering all activity intensities. Further investigation of school days separately was undertaken for MVPA only. MVPA was used as the outcome in these analyses as it is the intensity specifically targeted in UK government guidelines, and has been shown to be significantly associated with anthropometric measures in this sample [20]. However, as a sensitivity analysis these models were also run using cpm, producing the same patterns (results not shown). In order to assess if relationships varied by activity timing, separate models were run for school days only, and for specific parts of the school day equating to travel time (8 am-9 am and 3 pm-4 pm), lunchtime (12noon-2 pm) and the after school period (4 pm-9 pm). All analyses were undertaken in MLwiN [21] in 2014.

### Ethics approval

University of East Anglia Ethics Committee.

## Results

Of the 2031 participants originally recruited to the main SPEEDY study, 1019 took part in the second follow-up, and 480 in the third. From these, 281 (14% of the original sample) provided at least one valid school day of physical activity measurements on all three measurement occasions. Participating children provided 4047 valid physical activity measurement days, and attended 76 primary schools and 45 secondary schools.

Table 1 summarizes the characteristics of the participants included. As reported previously [14] participants generally became less active and more sedentary as they got older. Considering baseline characteristics, those included in these analyses were of a higher socio economic status (a higher proportion of households with more than 1 car, and lower proportion with a mother leaving fulltime education at < =16), and spent more time in VPA (both p < 0.05) compared to the excluded children. No statistically significant differences were observed between included and excluded children in terms of MVPA, sedentary time, cpm, BMI, weight status, age, gender or urban/rural location.

**Table 1:** **Characteristics of participants**

	**Number (%), mean (** ***standard deviation*** **), or Median, 25th centile-75th centile**		
	**9-10 years (2007)**	**10-11 years (2008)**	**13-14 years (2011)**
Sex (Female)	156 (55.1%)	-	-
Age	10.3 (*0.3*)	11.2 (*0.3*)	14.3 (*0.3*)
BMI	17.3, 15.9 - 19.2	-	20.1, 18.3 - 22.9
Weight status^a^			
Healthy weight	229 (81.5%)	-	228 (81.1%)
Overweight	44 (15.7%)	-	41 (14.6%)
Obese	8 (2.9%)	-	12 (4.3%)
Age parent left full time education^b^*			
<=16 years	103 (37.5%)	-	-
16-18 years	107 (38.9%)	-	-
>18 years	65 (23.6%)	-	-
Location^c^			
Urban	101 (36.2%)	-	-
Town & Fringe	74 (26.5%)	-	-
Rural	104 (37.2%)	-	-
Average daily time spent…			
Sedentary	471.5, 436 - 498.7	478.3, 449.5 - 508	513.8, 475.2 - 545
MVPA	73, 58.5 - 90.8	69.6, 54 - 86.1	59.8, 47.3 - 76.6
Vigorous or very vigorous*	24.2, 17 - 34.2	21.9, 15.2 - 30.4	16.7, 9.8 - 25.2
Registered time*	727.1 (*55.2*)	725.5 (*52.2*)	715.1 (*65.8*)
Average daily counts per minutes	611.3, 505.2 - 753.2	571.4, 462.8 - 680	452.7, 364 - 564.1
Number of valid days	6, 5 - 6	7, 6 - 7	6, 5 - 7

^a^weight status missing for 2 participants, ^b^age parent left full time education missing for 8 participants, ^c^location missing for 4 participants. *At SPEEDY 1 those included in analysis significantly different (p < 0.05) to those not included.

**Figure 1:**
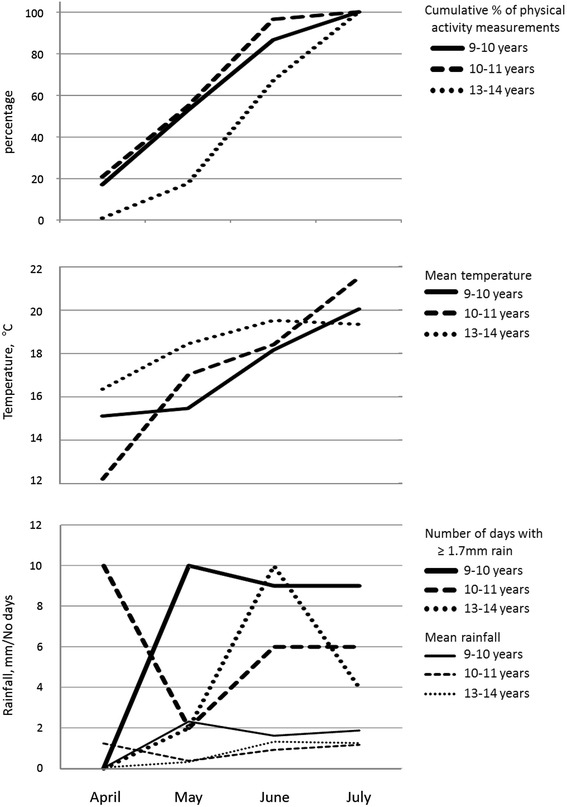
**Summary of physical activity measurement dates, temperature and rainfall by month over the three data collection periods; ages 9-10 years (2007), 10-11 years (2008) and 13-14 years (2011).**

Figure 1 summarizes monthly temperature, rainfall and physical activity measurement dates for the three study periods. This shows that a high proportion of physical activity measurements at 9–10 years and 10–11 years were undertaken in April and May, which in both years were cooler and wetter. At 13–14 years, a higher proportion of measurements were undertaken later in the summer (June and July), which was warmer, but also relatively wet. The distribution of measurement days falling into each rainfall category at each study phase are shown in Table 2. A Chi^2^ test indicated no significant association between study phase and rainfall category, suggesting a relatively even distribution of the rainfall categories between the three data collection periods.

**Table 2:** **Distribution of rainfall categories across the three study phases**

	**Daily rainfall categories (mm)**			
	**0**	**>0 - <1.7**	**≥1.7**	**All categories**
9-10 years (2007)	39 (43.8%)	23 (25.8%)	27 (30.3%)	89 (100%)
10-11 years (2008)	61 (55%)	29 (26.1%)	21 (18.9%)	111 (100%)
13-14 years (2011)	53 (59.6%)	20 (22.5%)	16 (18%)	89 (100%)
All phases	153 (52.9%)	72 (24.9%)	64 (22.1%)	289 (100%)

Chi^2^ test for association between study phase and rainfall category p = 0.144.

**Figure 2:**
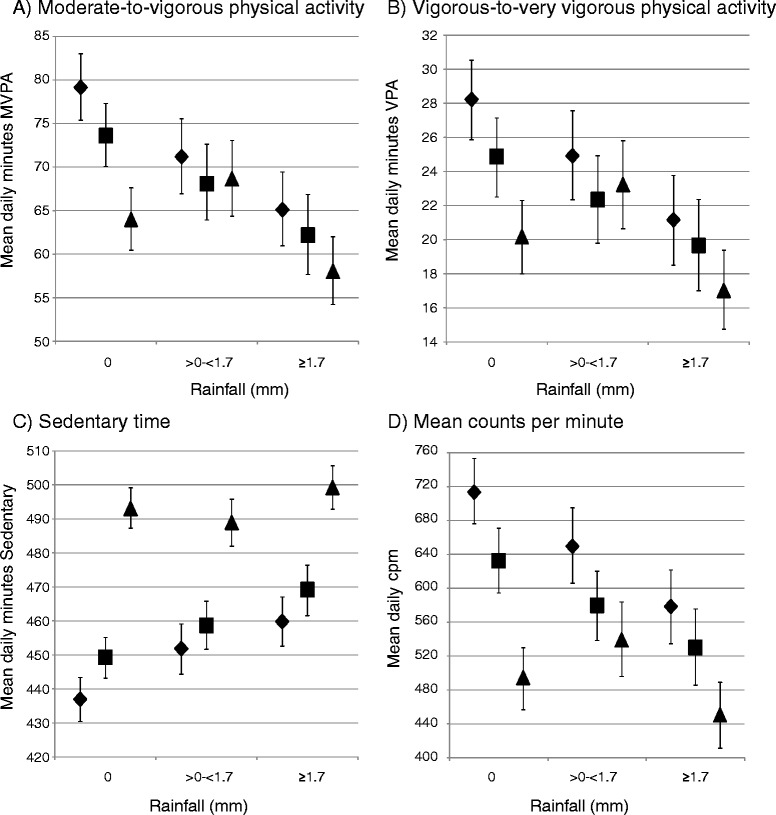
**Adjusted** ^**1**^ **mean (with 95% confidence intervals) physical activity outcomes over all days (weekend and week day) by study phase and rainfall tertile. A)** Moderate-to-vigorous physical activity, **B)** Vigorous-to-very-vigorous physical activity, **C)** Sedentary time, **D)** Mean counts per minute. ♦ 9-10 years, ■ 10-11 years, ▲ 13-14 years. ^1^ Adjusted for age, sex, accelerometer wear time (for SED, MVPA and VPA models), mean daily temperature and day type (weekend/week day).

Figure 2 shows adjusted mean daily physical activity for each study phase and rainfall category. Rainfall was significantly associated with all physical activity outcomes at 9–10 years and 10–11 years whereby MVPA, VPA and cpm all declined and sedentary time increased with increased rainfall. However, the same patterns were not seen at 13–14 years. At 13–14 years activity, was generally lower and inactivity higher, yet there was no significant trend across rainfall categories. The resulting patterns therefore show statistically significant declines between study phases on dry days only (except cpm which is significantly lower at 13–14 years than 9–10 years in all rainfall categories). At 13–14 years sedentary time was statistically significantly higher than at 9–10 years and 10–11 years across all rainfall categories. Results from the regression models are presented in Additional file 1: Table S1.

**Figure 3:**
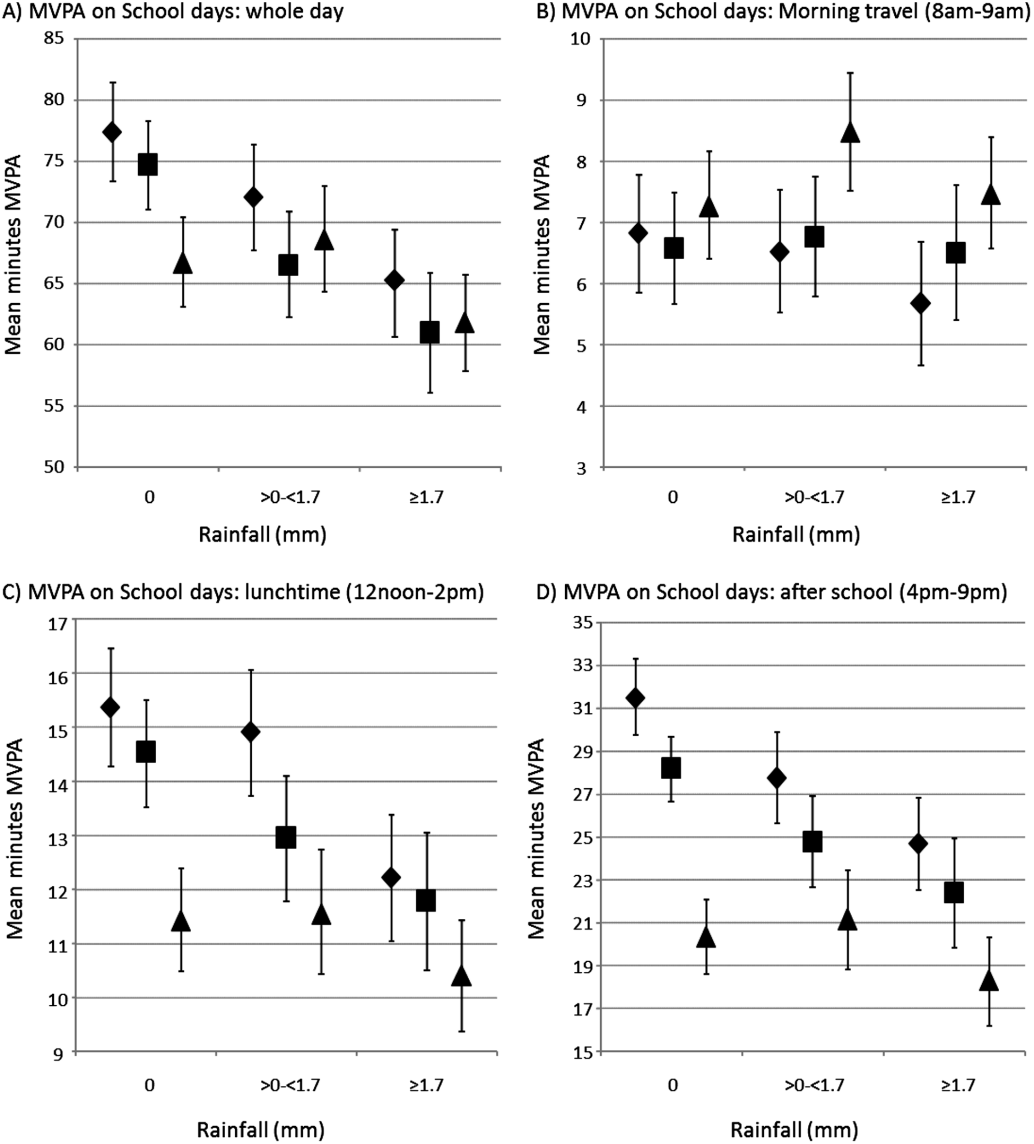
**Adjusted** ^**1**^ **mean (with 95% confidence intervals) MVPA on school days by study phase and rainfall tertile. A)** Whole day, **B)** Morning travel time (8am-9am), **C)** Lunchtime (12noon-2pm), **D)** After school (4pm-9pm). ♦ 9-10 years, ■ 10-11 years, ▲ 13-14 years. ^1^ Adjusted for age, sex, accelerometer wear time, and mean daily temperature.

Figure 3 shows the adjusted mean MVPA for school days only, considering the whole day, and specific periods of the day; 8 am-9 am (the morning commute; afternoon commute excluded for brevity, but similar pattern seen), 12noon-2 pm (the lunchtime period), and 4 pm-9 pm (the after school period). The whole day results show a similar pattern to that seen when all days were considered; clear, statistically significant decreases in MVPA with increasing rainfall at 9–10 years and 10–11 years, but no trend at 13–14 years along with generally lower levels of MVPA at later phases.

The observed pattern was particularly pronounced in the lunchtime and after school periods. At 9–10 years, children undertook an average of 22% (6.8 minutes) less MVPA on the wettest days relative to the driest days during the after school period. The differences between 9–10 years and 13–14 years were 11.1 minutes on the driest days and 6.4 minutes on the wettest. These patterns were not seen so clearly during the commuting period. Here the trend of decreased activity with increasing rainfall was not apparent at any study phase, and while the difference was not statistically significant, MVPA during this period was slightly higher at 13–14 years. Results from the regression models are presented in Additional file 2: Table S2.

## Discussion

We investigated the relationship between rainfall and physical activity among a cohort of English schoolchildren at ages 9–10 years, 10–11 years and 13–14 years. A statistically significant trend of decreasing activity (MVPA, cpm and VPA) and increasing sedentary time was observed with increased rainfall at ages 9–10 years and 10–11 years, but not at 13–14 years. These patterns were apparent when the whole day was considered, and during specific parts of the school day; during lunchtime (12noon-2 pm) and after school (4 pm-9 pm). However they were not seen during school commute times (8-9 am and 3-4 pm).

Taken together, our findings suggest that rainfall has a greater impact during periods when children have more freedom (e.g. lunchtime and after school) compared to times when their activity is more habitualised (e.g. travel to school). This could suggest that rainfall impacts certain types of activity (e.g. free play) more than others (active travel). It is possible that a shift away from free play-type activities, such as skipping [22], as children age is the reason for the lack of association between rainfall and activity at ages 13–14 years.

Although activity levels have been seen to decline with age in this cohort [14], this pattern was not seen so clearly on the wettest days. It is possible that the MVPA and VPA children undertake on wet days represents a baseline of relatively fixed components that are maintained despite adverse weather conditions, and are thus less susceptible to age-related declines. Identifying the sources of MVPA and VPA on wet days may provide insights into sustainable types of activities that could promote ongoing activity as children age. In line with this suggestion, our findings for commuting periods of the school day serve to re-emphasise the value of active travel for physical activity promotion. It is during this period that no significant decline in activity was seen with increasing rainfall or age. This highlights the potential importance of habitualising behavior to make children more resilient both to bad weather, and potentially age-related decline in activity.

We have previously suggested that interventions to increase physical activity in primary school children should consider the impact of rainfall [12], and be designed to work well in all conditions. Strategies specifically targeting activity promotion on wet days may help increase overall activity among primary school children. However, given the lack of associations seen between rainfall and activity, and the overall low levels of MVPA at ages 13–14 years, such considerations may be less relevant for interventions in secondary schools, where general physical activity promotion strategies may be more appropriate.

While seasonality in children’s physical activity is well recognized, and longitudinal studies often take steps to adjust for it in both data collection procedures and statistical analyses, little consideration has been given to the impact of weather conditions on the longitudinal assessment of children’s physical activity. Of the 26 papers included in the systematic review and pooled analysis of change in children’s physical activity by Dumith et al. [23], only one investigated the impact of season and weather [3], and even that did not consider their impact on *change* in physical activity. Our findings suggest that consideration of the weather conditions experienced during physical activity data collection could be important, especially given the differing impact they have on older and younger children.

This study has a number of strengths and weaknesses. In terms of strengths, we were able to analyze repeated longitudinal measurements from the same cohort of children. We also used repeated objective measures of both physical activity and rainfall. This allowed us to examine in detail activity of differing intensities at specific points in the day, and gave variation in rainfall exposure within subjects as they aged.

The study’s weaknesses include the restricted period over which data were collected; between mid-April to mid-July. This gave the potential for participants to be exposed to quite different rainfall conditions at each phase. Ultimately, although weather conditions did differ between the three periods, all three years were wetter than long-term averages [24], providing a relatively even distribution of rainfall conditions across the phases, and making it possible to compare the effects of similar conditions at different participant ages. However, while all three of the study years saw a mixture of wet and dry weather, we cannot say whether the associations we observed would be seen at other times of the year, nor begin to disentangle seasonal and weather effects. The restricted measurement periods also meant that the temperatures participants were exposed to were relatively benign, with 95% of days being in the range 10.3 to 25.1°C. Sampling over a longer period would be necessary to investigate relationships between temperature and physical activity, and if and how these change as children age.

Participants provided up to six days of activity data at each phase, but these were over consecutive days, potentially limiting the variability of rainfall each participant was exposed to. Our rainfall data were from an official source and were available at hourly intervals, however, they were only from two locations. While there was strong correlation between the two stations, giving us confidence that the daily rain totals we derived from them were broadly representative of the daily rainfall for each school, some miss-classification of rainfall exposure was inevitable, potentially attenuating the associations seen. The mean distance for primary schools to nearest weather station was 29 km (SD:14 km), and so we did not attempt to measure whether it was actually raining during specific periods of the school day.

Accelerometers provide an objective measure of physical activity, but they are not without their limitations. They have a limited ability to assess activity while the wearer is cycling [25], and must be removed altogether during aquatic activities. Under-detection of cycling-related activity may have impacted the relationship seen at commuting times, and could have induced a bias if cyclists were likely to change mode on wet days. However, we consider this unlikely given the low numbers usual cyclists in our sample (11% at 9–10 years, 8% at 10–11 years and 4% at 13–14 years). A sensitivity test of commuting time models without the participants who reported usually cycling to school produced almost identical results to those presented in Figure 3. Due to the small number of cyclists in this sample, it is important to note that the results presented in this manuscript are potentially more valid for those using other modes of transport to school. A final limitation is the generalizability of our findings. Schools in Norfolk, and consequently our sample, have a low proportion of non-white pupils, which potentially limits the generalizability of our findings to more ethnically diverse populations. Additionally, compared to those excluded, those included in our sample tended to be of a higher socio-economic status and recorded significantly more VPA at baseline. Although there is no evidence that weather conditions may influence these children differently, generalisation of these results to less active children and those of lower socio-economic status should be made with caution.

To conclude, we found that increased rainfall was associated with significant decreases in MVPA, VPA, overall activity (cpm), and increases in sedentary time among primary school children, but not secondary school children. Activity declines most steeply between the ages of 9–10 years and 13–14 years on dry days. We suggest it is important to consider weather conditions experienced during physical activity data collection, and interventions to increase activity on wet days in primary schools may help increase overall activity levels in children.

## Additional files

Additional file 1: Table S1. Results of multilevel regression models of daily physical activity and sedentary time by rainfall category. Additional file 2: Table S2. Results of multilevel regression models of moderate-to-vigorous physical activity in different periods of the school day.

## References

[1] Fisher A, Reilly JJ, Montgomery C, Kelly LA, Williamson A, Jackson DM (2005). Seasonality in physical activity and sedentary behavior in young children. Pediatr Exerc Sci.

[2] Riddoch CJ, Mattocks C, Deere K, Saunders J, Kirkby J, Tilling K (2007). Objective measurement of levels and patterns of physical activity. Arch Dis Child.

[3] Bélanger M, Gray-Donald K, O’Loughlin J, Paradis G, Hanley J (2009). Influence of weather conditions and season on physical activity in adolescents. Ann Epidemiol.

[4] Hjorth MF, Chaput J, Michaelsen K, Astrup A, Tetens I, Sjödin A (2013). Seasonal variation in objectively measured physical activity, sedentary time, cardio-respiratory fitness and sleep duration among 8–11 year-old Danish children: a repeated-measures study. BMC Public Health.

[5] Gracia-Marco L, Ortega FB, Ruiz JR, Williams CA, Hagströmer M, Manios Y (2013). Seasonal variation in physical activity and sedentary time in different European regions. The HELENA study. J Sports Sci.

[6] Rich C, Griffiths LJ, Dezateux C (2012). Seasonal variation in accelerometer-determined sedentary behaviour and physical activity in children: a review. Int J Behav Nutr Phys Act.

[7] Chan CB, Ryan DA (2009). Assessing the effects of weather conditions on physical activity participation using objective measures. Int J Environ Res Public Health.

[8] Brockman R, Jago R, Fox KR (2011). Children’s active play: self-reported motivators, barriers and facilitators. BMC Public Health.

[9] Cooper AR, Page AS, Wheeler BW, Hillsdon M, Griew P, Jago R (2010). Patterns of GPS measured time outdoors after school and objective physical activity in English children: the PEACH project. Int J Behav Nutr Phys Act.

[10] Ridgers ND, Stratton G, Fairclough SJ (2006). Physical activity levels of children during school playtime. Sports Med.

[11] Harrison F, Jones AP (2012). A framework for understanding school based physical environmental influences on childhood obesity. Health Place.

[12] Harrison F, Jones AP, Bentham G, van Sluijs EMF, Cassidy A, Griffin SJ (2011). The impact of rainfall and school break time policies on physical activity in 9–10 year old British children: a repeated measures study. Int J Behav Nutr Phys Activ.

[13] van Sluijs EMF, Skidmore PML, Mwanza K, Jones AP, Callaghan AM, Ekelund U (2008). Physical activity and dietary behaviour in a population-based sample of British 10-year old children: the SPEEDY study (Sport, Physical activity and Eating behaviour: Environmental Determinants in Young people). BMC Public Health.

[14] Corder K, Sharp SJ, Atkin AJ, Griffin SJ, Jones AP, et al. Change in objectively measured physical activity during the transition to adolescence. Br J Sports Med 2014. [Epub ahead of print]. 10.1136/bjsports-2013-093190 PMC4453714 24273308

[15] Bibby P, Shepherd J (2004). Developing a new classification of urban and rural areas for policy purposes - The methodology.

[16] Ekelund U, Åman J, Westerterp K (2003). Is the ArteACC index a valid indicator of free-living physical activity in adolescents?. Obes Res.

[17] Corder K, van Sluijs EMF, Ekelund U, Jones AP, Griffin SG (2010). Changes in children’s physical activity over 12 months: longitudinal results from the SPEEDY study. Pediatrics.

[18] Riddoch CJ, Bo Andersen L, Wedderkopp N, Harro M, Klasson-Heggebo L, Sardinha LB (2004). Physical activity levels and patterns of 9- and 15-yr-old european children. Med Sci Sports Exerc.

[19] MIDAS Land Surface Stations data (1853-current). [http://badc.nerc.ac.uk/view/badc.nerc.ac.uk__ATOM__dataent_ukmo-midas]

[20] Steele RM, van Sluijs EMF, Cassidy A, Griffin SJ, Ekelund U (2009). Targeting sedentary time or moderate- and vigorous-intensity activity: independent relations with adiposity in a population-based sample of 10-y-old British children. Am J Clin Nutr.

[21] Rasbash J, Browne WJ, Healy M, Cameron B, Charlton C (2012). MLwiN Version 2.26.

[22] Brooke HL, Corder K, Griffin SG, van Sluijs EMF (2014). Physical activity maintenance in the transition to adolescence: a longitudinal study of the roles of sport and lifestyle activities in British youth. PLoS One.

[23] Dumith SC, Gigante DP, Domingues MR, Kohl HW (2011). Physical activity change during adolescence: a systematic review and a pooled analysis. Int J Epidemiol.

[24] Eastern England: climate. [http://www.metoffice.gov.uk/climate/uk/ee/]

[25] Sirard JR, Pate RR (2001). Physical activity assessment in children and adolescents. Sports Med.

