# Supplementary material for: The changing relationship between rainfall and children’s physical activity in spring and summer: a longitudinal study
Source: Int J Behav Nutr Phys Act. 2015 Mar 21;12:41. doi: 10.1186/s12966-015-0202-8 (PMC4377032; doi:10.1186/s12966-015-0202-8)
Supplement: Additional file 1: Table S1. — Results of multilevel regression models of daily physical activity and sedentary time by rainfall category. [file 12966_2015_202_MOESM1_ESM.pdf]

Supplementary Table 1. Results of multilevel regression models of daily physical activity and sedentary time by rainfall category.

	MVPA			VPA			Sedentary time			counts per minute		
	β	S.E.	p	β	S.E.	p	β	S.E.	p	β	S.E.	p
Age (years)	2.387	3.41	*0.484*	0.467	1.952	*0.811*	2.761	5.725	*0.629*	-12.697	32.082	*0.692*
Sex (female)	-12.915	1.988	**<0.001**	-8.631	1.18	**<0.001**	18.997	3.452	**<0.001**	-87.404	19.524	**<0.001**
Weekend day	3.262	0.956	**0.001**	1.245	0.521	0.017	-17.529	1.561	**<0.001**	56.194	8.753	**<0.001**
Registered time (minutes)	0.115	0.006	**<0.001**	0.041	0.003	**<0.001**	0.615	0.009	**<0.001**			
Daytime temperature (°C)	0.147	0.145	*0.313*	-0.143	0.079	*0.070*	-0.708	0.234	**0.003**	1.892	1.397	*0.176*
Study Phase												
SPEEDY 2 (2008)	-7.868	3.678	0.032	-3.807	2.083	*0.068*	9.53	6.125	*0.120*	-67.882	34.706	*0.050*
SPEEDY 3 (2011)	-24.877	13.97	*0.075*	-9.93	7.994	*0.214*	44.966	23.421	*0.055*	-167.54	131.278	*0.202*
Rainfall tertile												
T2: >0 - <1.7mm	-7.955	1.883	**<0.001**	-3.269	1.022	**0.001**	14.875	3.055	**<0.001**	-63.413	18.009	**<0.001**
T3: ≥1.7mm	-14.008	1.872	**<0.001**	-7.056	1.018	**<0.001**	22.81	3.04	**<0.001**	-134.434	18.064	**<0.001**
Interactions												
Rainfall T2 # SPEEDY 2	2.43	2.538	*0.338*	0.77	1.373	*0.575*	-5.373	4.103	*0.190*	9.817	24.047	*0.683*
Rainfall T3 # SPEEDY 2	2.576	2.62	*0.325*	1.857	1.437	*0.196*	-2.94	4.293	*0.493*	32.365	25.789	*0.209*
Rainfall T2 # SPEEDY 3	12.691	2.744	**<0.001**	6.298	1.488	**<0.001**	-19.079	4.419	**<0.001**	108.104	26.299	**<0.001**
Rainfall T3 # SPEEDY 3	8.139	2.608	**0.002**	3.904	1.407	**0.006**	-16.674	4.196	0.016	91.154	24.844	**<0.001**
Variance Partition Coefficients												
School	11.6%		**0.008**	17.1%		**0.008**	11.1%		**0.008**	14.5%		**0.008**
Participant	19.4%		**<0.001**	19.8%		**<0.001**	21.3%		**<0.001**	18.0%		**<0.001**
Day	69.1%		**<0.001**	63.1%		**<0.001**	67.6%		**<0.001**	67.5%		**<0.001**

Reference categories for categorical variables: Sex = male, Weekend day = week day, Study phase = SPEEDY 1 (2007), Rainfall tertile = Tertile 1 (0mm rain).

Variance Partition Coefficients describe the percentage of residual variance remaining at each level of the model hierarchy.

For p values, **bold font** indicates statistical significance p<0.01, regular font indicates statistical significance p<0.05, and *italic font* indicates statistical non-significance (p≥0.05)
