# Supplementary material for: The changing relationship between rainfall and children’s physical activity in spring and summer: a longitudinal study
Source: Int J Behav Nutr Phys Act. 2015 Mar 21;12:41. doi: 10.1186/s12966-015-0202-8 (PMC4377032; doi:10.1186/s12966-015-0202-8)
Supplement: Additional file 2: Table S2. — Results of multilevel regression models of moderate-to-vigorous physical activity in different periods of the school day. [file 12966_2015_202_MOESM2_ESM.pdf]

Supplementary Table 2. Results of multilevel regression models of moderate-to-vigorous physical activity in different periods of the school day.

	All day (7am-9pm)			Morning Travel (8am-9am)			Lunchtime (12noon-2pm)			After school (4pm-9pm)		
	β	S.E.	p	β	S.E.	p	β	S.E.	p	β	S.E.	p
Age (years)	3.245	3.452	*0.347*	1.881	0.714	**0.008**	0.159	0.781	*0.840*	0.013	1.757	*1.000*
Sex (female)	-14.78	2.102	**<0.001**	-0.491	0.438	*0.262*	-5.678	0.473	**<0.001**	-4.892	1.054	**<0.001**
Registered time (minutes)	0.11	0.006	**<0.001**	0.144	0.005	**<0.001**	0.103	0.011	**<0.001**	0.105	0.098	**<0.001**
Daytime temperature (°C)	-0.062	0.153	*0.686*	-0.013	0.03	*0.649*	-0.095	0.043	0.025	0.126	0.005	*0.285*
Study Phase												
SPEEDY 2 (2008)	-5.903	3.82	*0.122*	-2.116	0.774	**0.006**	-0.96	0.882	*0.276*	-3.291	2.042	*0.107*
SPEEDY 3 (2011)	-23.82	14.157	*0.092*	-7.194	2.931	0.014	-4.578	3.215	*0.154*	-11.155	7.213	*0.122*
Rainfall tertile												
T2: >0 - <1.7mm	-5.409	1.898	**0.004**	-0.322	0.363	*0.375*	-0.441	0.522	*0.398*	-3.685	1.256	**0.003**
T3: ≥1.7mm	-12.159	2.04	**<0.001**	-1.143	0.391	**0.003**	-3.15	0.549	**<0.001**	-6.785	1.314	**<0.001**
Interactions												
Rainfall T2 # SPEEDY 2	-2.846	2.57	*0.268*	0.498	0.492	*0.312*	-1.153	0.711	*0.105*	0.268	1.708	*0.874*
Rainfall T3 # SPEEDY 2	-1.516	2.975	*0.610*	1.071	0.57	*0.060*	0.41	0.802	*0.609*	1.017	1.915	*0.595*
Rainfall T2 # SPEEDY 3	7.293	2.728	**0.008**	1.534	0.525	**0.003**	0.569	0.749	*0.447*	4.503	1.791	0.012
Rainfall T3 # SPEEDY 3	7.239	2.706	**0.007**	1.347	0.523	0.010	2.157	0.742	**0.004**	4.745	1.779	**0.008**
Variance Partition Coefficients												
School	14.6%		**0.004**	29.4%		**<0.001**	16.9%		**<0.001**	0.5%		*0.599*
Participant	23.0%		**<0.001**	21.3%		**<0.001**	14.6%		**<0.001**	17.7%		**<0.001**
Day	62.4%		**<0.001**	49.4%		**<0.001**	68.6%		**<0.001**	81.9%		**<0.001**

Reference categories for categorical variables: Sex = male, Study phase = SPEEDY 1 (2007), Rainfall tertile = Tertile 1 (0mm rain)

Variance Partition Coefficients describe the percentage of residual variance remaining at each level of the model hierarchy.

For p values, **bold font** indicates statistical significance p<0.01, regular font indicates statistical significance p<0.05, and *italic font* indicates statistical non-significance (p≥0.05)
